# Matrix Metalloproteases and Tissue Inhibitors of Metalloproteinases in Medial Plica and Pannus-like Tissue Contribute to Knee Osteoarthritis Progression

**DOI:** 10.1371/journal.pone.0079662

**Published:** 2013-11-04

**Authors:** Chih-Chang Yang, Cheng-Yu Lin, Hwai-Shi Wang, Shaw-Ruey Lyu

**Affiliations:** 1 Department of Anatomy, National Yang-Ming University, Taipei, Taiwan, R.O.C.; 2 Joint Center, Tzu-Chi Dalin General Hospital, Chiayi, Taiwan, R.O.C.; 3 Tzu-Chi University, Hualien, Taiwan, R.O.C.; Van Andel Institute, United States of America

## Abstract

Osteoarthritis (OA) is characterized by degradation of the cartilage matrix, leading to pathologic changes in the joints. However, the pathogenic effects of synovial tissue inflammation on OA knees are not clear. To investigate whether the inflammation caused by the medial plica is involved in the pathogenesis of osteoarthritis, we examined the expression of matrix metalloproteinases (MMPs), tissue inhibitors of metalloproteinases (TIMPs), interleukin (IL)-1β, and tumor necrosis factor (TNF)-α in the medial plica and pannus-like tissue in the knees of patients with medial compartment OA who underwent either arthroscopic medial release (stage II; 15 knee joints from 15 patients) or total knee replacement (stage IV; 18 knee joints from 18 patients). MMP-2, MMP-3, MMP-9, IL-1β, and TNF-α mRNA and protein levels measured, respectively, by quantitative real-time PCR and Quantibody human MMP arrays, were highly expressed in extracts of medial plica and pannus-like tissue from stage IV knee joints. Immunohistochemical staining also demonstrated high expression of MMP-2, MMP-3, and MMP-9 in plica and pannus-like tissue of stage IV OA knees and not in normal cartilage. Some TIMP/MMP ratios decreased significantly in both medial plica and pannus-like tissue as disease progressed from stage II to stage IV. Furthermore, the migration of cells from the pannus-like tissue was enhanced by IL-1β, while plica cell migration was enhanced by TNF-α. The results suggest that medial plica and pannus-like tissue may be involved in the process of cartilage degradation in medial compartment OA of the knee.

## Introduction

Osteoarthritis (OA) is characterized by degradation of the cartilage matrix and gradually progresses without any repair of the damaged tissue, leading to pathologic changes in the joints. Clinical symptoms in the OA knee include joint pain, inflammation, and functional disability of the joints. Previous studies on patients with OA of the knee have focused on degradation of the cartilage extracellular matrix [1-4]. More recently, synovial tissue inflammation was also found to be a pathogenetic factor in the OA knee [5-8].

The mediopatellar (medial) plica, an embryonic remnant in the synovial cavity of the knee [9,10], can cause knee pain and can be removed by arthroscopic resection for symptom relief [11-14] and this might also modify the disease process [15]. Since various degrees of cartilage degeneration on the surface of the medial femoral condyle facing the medial plica have been observed [14,16-19], a series of studies on medial plica-related abrasion phenomenon were performed and provided evidence for a role of pathologic medial plica in the pathogenesis of medial compartment OA of the knee joint [15,20,21]. 

Pannus-like tissue shows dense vascularity and contains aggressive macrophage-like cells and invasive fibroblast-like cells. These cells, which may originate from the bone marrow [22-24] or synovial membrane [22,25], might contribute to cartilage erosion. Pannus-like tissue has been observed around the margin of the cartilaginous lesion on the medial femoral condyle opposite the inflamed medial plica in OA knees with medial abrasion phenomenon [26,27]. It was recently demonstrated that matrix metalloproteinase (MMP)-3 mRNA and protein are highly expressed in the medial plica and pannus-like tissue in the knees of patients with early stage medial compartment OA and that interleukin-1β (IL-1β) treatment of cells isolated from these tissues increases MMP-3 mRNA levels [28]. 

MMPs are a family of endopeptidases that act extracellularly to degrade multiple substrates in the extracellular matrix (ECM). MMP-1, -8, -13, and -18 are collagenases, MMP-2 and -9 are gelatinases, and MMP-3, -7, -10, and -11 are stromelysins [29]. Tissue inhibitors of metalloproteinases (TIMPs)-1, -2, and -4 are specific inhibitors of MMP-2, -3, and -9 [30]. Overexpression of MMPs results in an imbalance between the activity of MMPs and TIMPs that can lead to a variety of pathological disorders [31,32]. In cartilage, the ECM consists of collagens, gelatin, matrix glycoproteins, and proteoglycan. Since MMPs can degrade the cartilage ECM, they are thought to be involved in ECM breakdown in osteoarthritis and rheumatoid arthritis. The role of MMP-3 in cartilage damage in OA has been demonstrated in an experimental model of arthritis [33]. MMP-3 protein is expressed in the synovium and the superficial zone of cartilage in the knee joints of OA patients [34], in the joint cavity in advanced rheumatoid arthritis patients [35], and in pannus-like tissue in OA patients [36]. IL-1β and TNF-α can induce both chondrocytes and synoviocytes to produce MMPs to degrade cartilage matrix in OA patients [2]. TIMPs, which can regulate ECM remodeling and the activities of growth factors and their receptors by inhibiting MMPs, have also been shown to be expressed in human cartilage [37-39]. 

IL-1β and TNF-α, members of the pro-inflammatory cytokines, are involved in a variety of cellular functions, including induction of cell migration [40,41]. IL-1β induces expression of MMP-2 and -9 to degrade the ECM, allowing transendothelial migration [42,43], while TNF-α increases melanoma cell migration by upregulating MMP-2 and -9 expression [41]. Upregulation of MMPs resulting in destruction of articular cartilage has been reported in rheumatoid arthritis [44]. Synovial cell migration had been observed in rheumatoid arthritis [45]. However, the roles of IL-1β and TNF-α in the MMP/TIMP balance in plica and pannus-like tissue and the effect of plica and pannus-like tissue cell migration in the OA knee have not been investigated. 

In this study, we examined MMP and TIMP mRNA and protein levels in medial plica and pannus-like tissue of the knee in patients with medial compartment OA using quantitative RT-PCR, MMP ELISA arrays, and immunohistochemical staining. The effects of IL-1β or TNF-α treatment of cells isolated from these tissues on MMP and TIMP expression and the expression of ECM-degrading enzymes and cell migration were also examined. On the basis of on our results, we postulate that pathologic medial plica and the related pannus-like tissue might play an important role in the pathogenesis of medial compartment knee OA.

## Materials and Methods

### Ethics Statement

This study was approved by the Research Ethics Committee of the Buddhist Dalin Tzu Chi General Hospital, which is certified by the Department of Health in Taiwan (IRB Approval Number: B09704022). Informed consent forms were signed by all patients participating in this study.

### Patients and specimens

Fifteen specimens of medial plica and pannus-like tissue were obtained from 15 patients with Kellgren and Lawrence grade II (stage II) medial compartment OA who underwent arthroscopic medial release [15] and another 18 specimens were obtained from 18 patients with grade IV medial compartment OA who underwent total knee arthroplasty. Normal human cartilage cells (Chondrocytes) were obtained from ScienCell Research Laboratories (ScienCell Res. Lab., Carlsbad, CA), normal human cartilage was obtained from Cybrdi, biomaterials provider (Cybrdi, Inc. MD). The dissected specimens were large enough to perform primary culture, quantitative RT-PCR, ELISA, and immunohistochemical staining studies. 

### Detection of MMP-related proteins in lysates of medial plica and pannus-like tissue using ELISA-based arrays

To test for 10 MMP-related proteins in lysates of human OA medial plica and pannus-like tissue, an ELISA-based method using Quantibody chips from RayBiotech (Norcross, GA) was used according to the manufacturer's protocol, allowing the simultaneous measurement of MMP-1, MMP-2, MMP-3, MMP-8, MMP-9, MMP-10, MMP-13, TIMP-1, TIMP-2, and TIMP-4 levels. To prepare plica and pannus tissue lysates, tissue samples from stage ii or stage iv OA knees were frozen in liquid nitrogen and suspended in T-PER® tissue protein extraction reagent (Pierce Biotechnology, IL, USA) and the suspension homogenized at 4°C, then the homogenate was centrifuged at 15000g for 10 min at 4°C and the supernatant retained. The arrays were blocked with blocking solution for 1 h at room temperature, then 100 μl of the supernatant was added to each well and the arrays incubated at 4 °C overnight. The samples were then decanted and the wells washed 5 times with phosphate-buffered saline (PBS), then detection antibody cocktail was added to each well and the plates incubated for 2 h at room temperature. After 5 washes with PBS, the supplied fluorescent dye labeled-streptavidin was added and the arrays incubated for 1 h at room temperature, washed 5 times with PBS, and the fluorescent signal measured on a GenePix Personal 4100A scanner (Molecular Devices Corporation, Sunnyvale, CA, USA). 

### Quantitative real-time PCR assays on medial plica and pannus-like tissue

Total RNA was extracted from plica and pannus using TriPure isolation reagent (Roche, Mannheim, Germany), then oligo-primed cDNA was synthesized using SuperScript III reverse transcriptase (Invitrogen, CA, USA). PCR was carried out on a Roche LightCycler detection system (Roche, Mannheim, Germany) using a Fast SYBR Green Master Mix (Roche, Mannheim, Germany) following the manufacturer’s instructions. The forward and reverse primers used were: 5’-GGAGTCAACGGATTTGGTCGTA-3’ and 5’-GGCAACAATATCCACTTTACCAGAGT-3’ for GADPH, 5’-GGACAAGCTGAGGAAGATGC-3’ and 5’-TCGTTATCCCATGTGTCGAA-3’ for IL-1β, 5’-AGCCCATGTAGCAAACC-3’ and 5’-TGAGGTACAGGCCCTCTGAT-3’ for TNF-α, 5’-GCACCCATTTACACCTACACCAA-3’ and 5’-AGAGCTCCTGAATGCCCTTGA-3’ for MMP-2, 5’-CCTGGTACCCACGGAACCT-3’ and 5’-AGGACAAAGCAGGATCACAGTTG-3’ for MMP-3, and 5’-GGACGATGCCTGCAACGT-3’ and 5’-ACAAATACAGCTGGTTCCCAATC-3’for MMP-9. 

### Immunohistochemical staining

Specimens (about 0.3 cm^3^) of medial plica (n=18) and pannus-like tissue (n=18) of stage IV OA pateints were frozen in Tissue-Tec OCT (Sakura, CA, USA) above liquid nitrogen. For testing, the frozen tissue blocks were brought to -20 °C and 5 µm sections cut with a cryostat (Leica, Mannheim, Germany) and placed on slides (Dako, CA, USA), fixed in HistoCHOICE (Amresco, Ohio, USA), washed for 3 x 5 min with PBS, then digested for 5 min at 37°C with 20 μg / ml of proteinase K (PROtech, Taiwan, ROC). They were then incubated for 2 h at room temperature with rabbit polyclonal antibodies against human MMP-2, MMP-3, or MMP-9 (Millipore, MA, USA) or normal rabbit IgG (Millipore, MA, USA) diluted in PBS, washed three times with PBS, and incubated for 1 h at room temperature with a 1:100 dilution in PBS of biotinylated goat anti-rabbit IgG antibodies and streptavidin-conjugated peroxidase (Millipore, MA, USA). Bound antibodies were then visualized using diaminobenzidine, the slides counterstained with hematoxylin, and staining assessed under a microscope.

### Culture of cells from plica or pannus-like tissue

The pannus-like tissue or plica was cut into small pieces and centrifuged at 250 *g* for 5 minutes at room temperature, then the pellet was washed with, and resuspended in, serum-free Dulbecco’s modified Eagle's medium (DMEM/F12, Grand Island, NY, USA). The suspension was centrifuged at 250 *g* for 5 minutes at room temperature and the pelleted cells treated with 0.2 mg/ml of collagenase for 16 h at 37°C, washed, and treated with 2.5% trypsin for 30 min at 37°C with agitation. The cells were then washed and cultured in DMEM supplemented with 10% fetal bovine serum (Sigma St. Louis, MO, USA), 10 μg/ml of heparin, 100 U/ml of penicillin, and 100 μg/ml of streptomycin in 5% CO_2_ in a 37°C incubator, with a medium change every 3 or 5 days**.**

### Measurement of MMP-2, MMP-3, MMP-9, TIMP-1, TIMP-2, and TIMP-4 release by cultured cells and the effect of IL-1β or TNF-α

Cells isolated from the pannus-like tissue and medial plica from stage IV OA knees were plated in 6-well dishes (1.4x 10 ^5^ cells/well), treated with 50 ng/ml of IL-1β or 100 ng/ml of TNF-α for 24 h, and the culture supernatants collected for measurement of MMP-2, MMP-3, MMP-9, TIMP-1, TIMP-2, and TIMP-4 levels using ELISA kits (R&D Systems, Minneapolis, Minnesota, USA) according to the manufacturer’s instructions.

### Cell migration assay and the effect of IL-1β or TNF-α

Cells from plica or pannus-like tissue (1.5x 10 ^4^ cells/well) were plated in 24-well 8 μm Transwell plates (Falcon, NJ, USA) and incubated in medium alone or containing 50 ng/ml of IL-1β or 100 ng/ml of TNF-α or mixtures of Il-1β and 100 ng/ml of IL-1 receptor antagonist (IL-1 RA) or TNF-α and 100 ng/ml of the TNF-α inhibitor Enbrel and allowed to migrate across the membrane for 24 h at 37°C, then the cells attached to the top side of the membrane were removed and the migrated cells on the bottom side fixed, stained using crystal violet, and quantified by the OD at 595 nm. 

### Statistical analysis

Student's t test was used to evaluate the significance of differences in the quantitative real-time PCR, ELISA, and migration assay data at the level of p < 0.05. Statistical analyses were performed using SPSS software (version 13.0).

## Results

### ELISA assay for MMPs and TIMPs in lysates of plica and pannus-like tissue from stage II and stage IV patients

**Figure 1:**
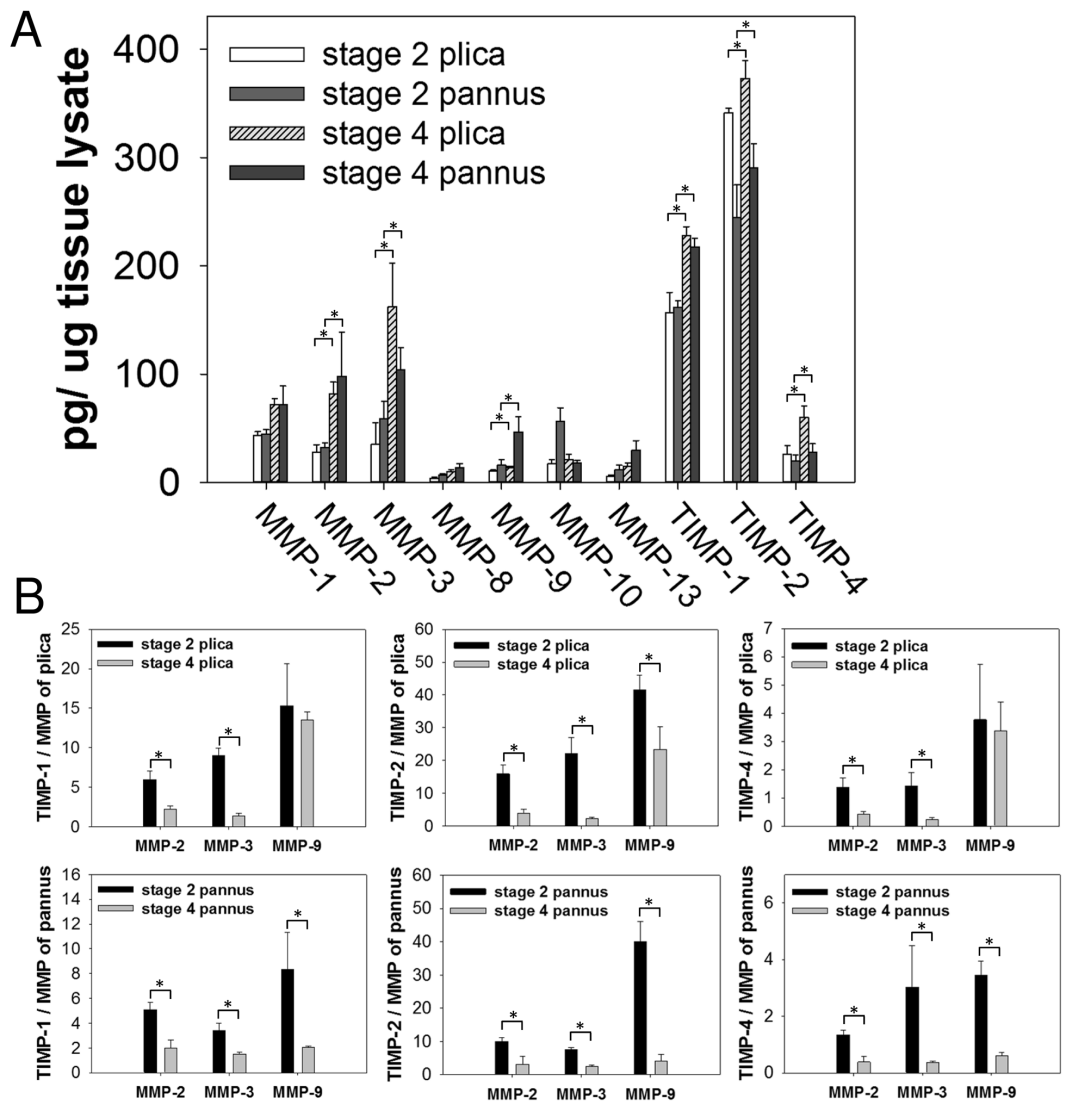
MMP and TIMP levels in lysates of medial plica and pannus-like tissue from OA patients. (A) Measurement of human MMP-1, MMP-2, MMP-3, MMP-8, MMP-9, MMP-10, MMP-13, TIMP-1, TIMP-2, and TIMP-4 using a Quantibody^®^ human MMP ELISA array. The data are the mean ± standard deviation (SD) (N = 15), *P < 0.05. (B) TIMP/MMP ratios in lysates of stage II and stage IV plica (upper panels) and pannus (lower panels) calculated from the Quantibody® human MMP array results. The data are the mean ± SD (N = 15), * P < 0.05.

As shown in Figure 1A, an ELISA array study showed that levels of MMP-2, MMP-3, MMP-9, TIMP-1, TIMP-2, and TIMP-4, but not MMP-1, MMP-8, MMP-10, or MMP-13, in lysates of plica and pannus-like tissue were significantly higher in stage IV OA than in stage II OA. Since enzyme/inhibitor imbalance may result in excessive matrix degradation, we compared the ratio of TIMP-1, TIMP-2, or TIMP-4 levels divided by MMP-2, MMP-3, or MMP-4 levels in lysates of the two tissues in both sets of OA patients. As shown in Figure 1B, TIMP-1/MMP-2, TIMP-2/MMP-2, TIMP-4/MMP-2, TIMP-1/MMP-3, TIMP-2/MMP-3, TIMP-4/MMP-3, and TIMP-2/MMP-9 ratios were all significantly higher in stage II OA than in stage IV in both the plica (Figure 1B, upper panels) and pannus-like tissue (Figure 1B, lower panels), while, in pannus-like tissue, the TIMP-1/MMP-9 and TIMP-4/MMP-9 ratios were also significantly higher in stage II OA than in stage IV (Figure 1B, lower panels).

### MMP and TIMP mRNA expression in plica and pannus-like tissue from stage IV OA patients

**Figure 2:**
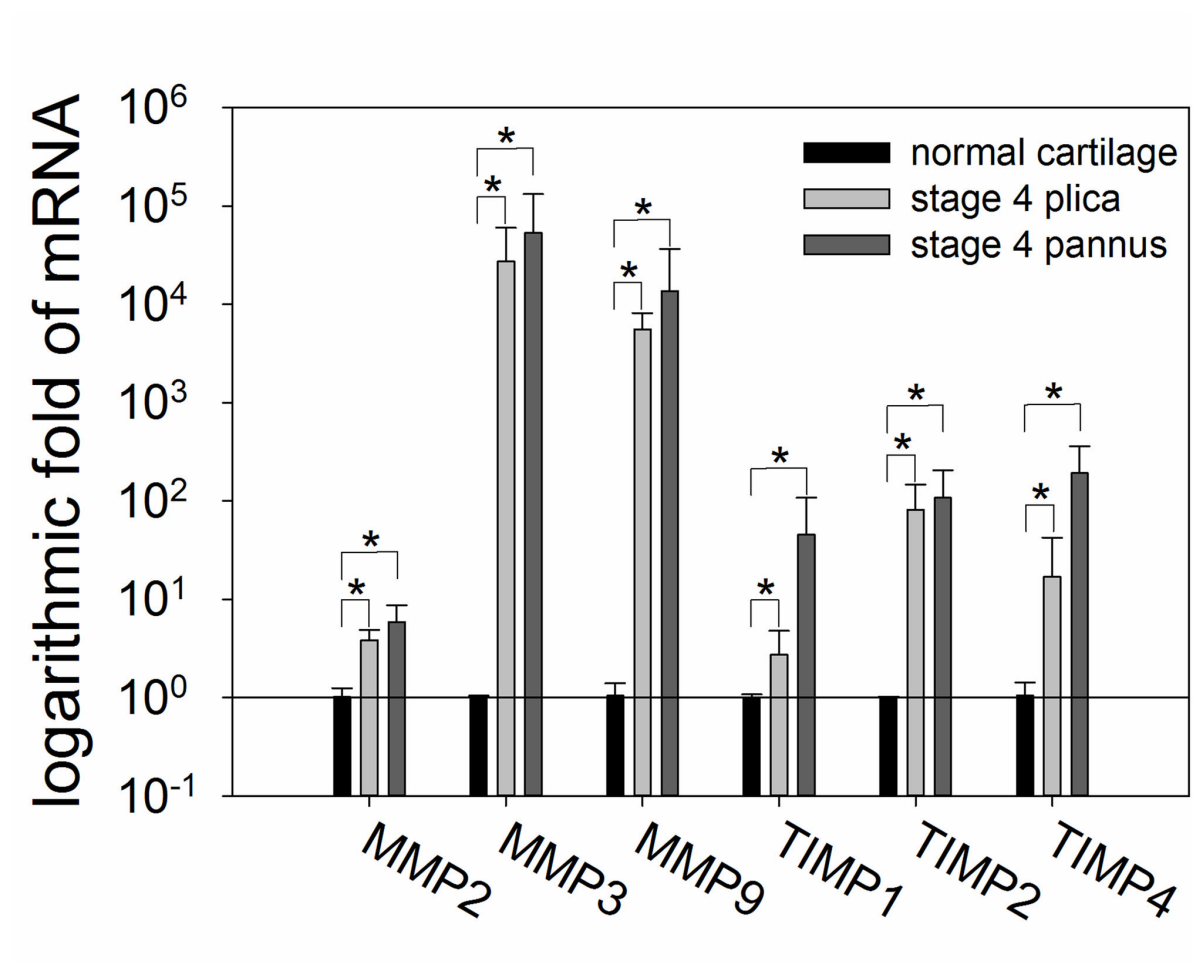
Real-time polymerase chain reaction measurement of mRNA levels for the indicated MMPs and TIMPs in medial plica and pannus-like tissue from knees from patients with stage IV OA and in normal cartilage. Total RNA was isolated from the medial plica and pannus-like tissue of stage IV OA knees (N = 15 for both) or normal cartilage (N = 3) and mRNA levels for the indicated MMPs and TIMPs measured by real-time PCR and expressed relative to that in normal cartilage. Each bar represents the mean ± standard deviation (SD), * P < 0.05.

Since the ELISA array data showed that MMP-2, MMP-3, MMP-9, TIMP-1, TIMP-2, and TIMP-4 proteins were highly expressed in plica and pannus-like tissues in stage IV patients (Figure 1A), we examined MMP and TIMP mRNA expression in these two tissues from stage IV OA patients and in normal cartilage by quantitative real-time PCR assay. As shown in Figure 2, MMP-2, MMP-3, MMP-9, TIMP-1, TIMP-2, and TIMP-4 mRNA levels were significantly higher in plica and pannus-like tissue from patients than in normal cartilage. 

### Immunohistochemical staining of plica and pannus-like tissue from stage IV patients for MMP-2, MMP-3, and MMP-9

**Figure 3:**
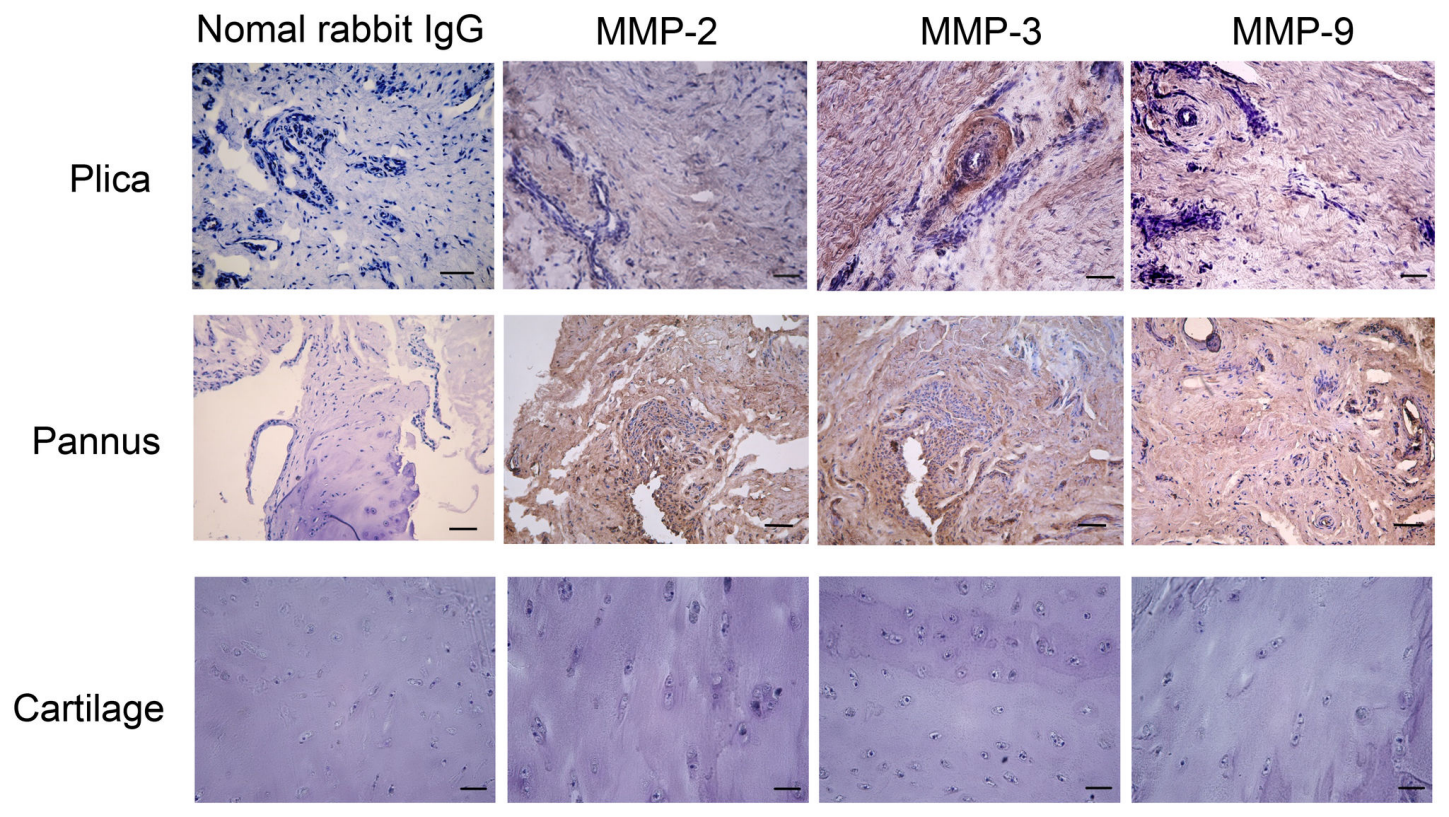
Immunohistochemical staining for MMP-2, MMP-3, or MMP-9 in the medial plica and pannus tissue of stage IV OA patients and normal cartilage. The panels on the left are negative controls using normal rabbit IgG instead of primary antibody. The bars represent 10 μm. The results are typical of those seen for all 18 OA knees.

Immunohistochemical staining was used to localize the expression of MMP-2, MMP-3, and MMP-9 in plica and pannus-like tissues from stage IV OA patients. As shown in Figure 3, all three proteins were highly expressed in plica and pannus-like tissues from stage IV OA patients, but not in normal cartilage. Staining using normal rabbit IgG instead of the primary antibody (negative control) is shown in the left column.

IL-1β and TNF-α mRNA levels in plica and pannus-like tissues and IL1-β- and TNF-α-induced release of MMPs and TIMPs by cultured cells isolated from plica and pannus-like tissue from stage IV OA patients

**Figure 4:**
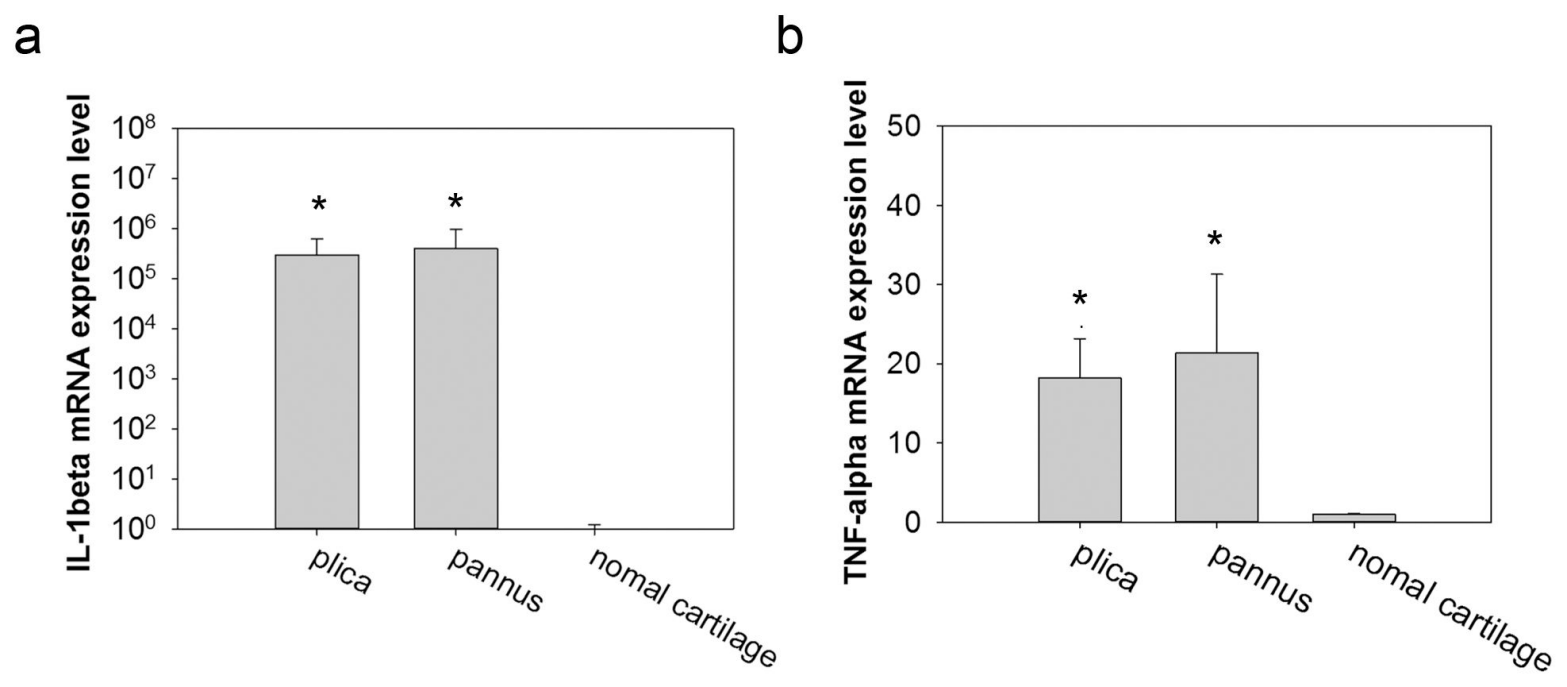
IL-1β and TNF-α mRNA levels in the medial plica and pannus-like tissue in stage IV OA knees and normal cartilage. Real-time polymerase chain reaction analysis of IL-1β (left panel) and TNF-α (right panel) mRNA levels in the medial plica and pannus-like tissue in stage IV osteoarthritis knees and normal cartilage expressed relative to levels in normal cartilage. Each bar represents the mean ± standard deviation for 15 samples. * P < 0.05.

Quantitative real-time PCR showed that IL-1β (Figure 4 a) and TNF-α (Figure 4 b) mRNAs were significantly and highly expressed in plica and pannus-like tissues from stage IV OA knees compared to normal cartilage.

**Figure 5:**
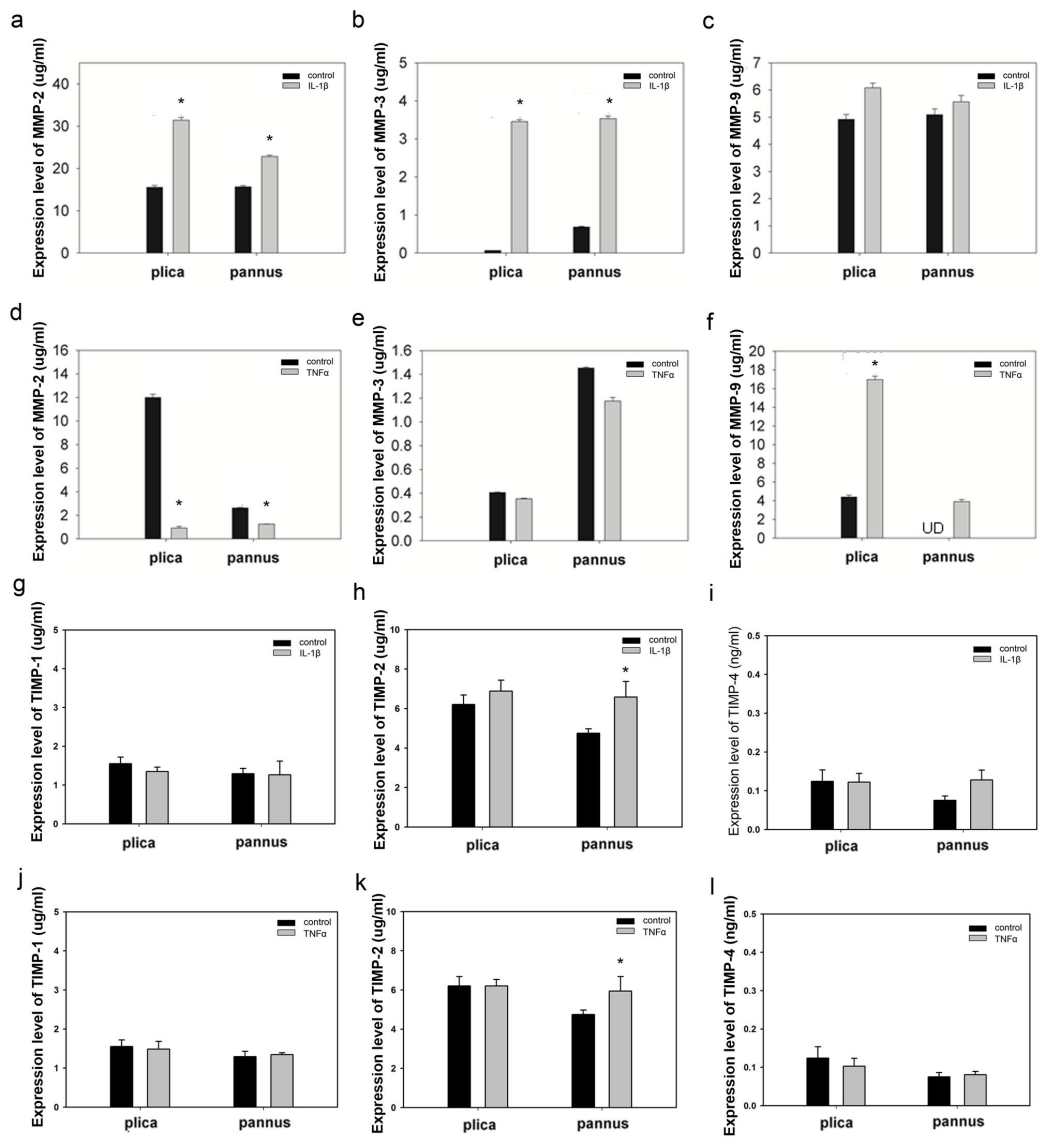
Effects of IL-1β and TNF-α on MMP secretion by cells isolated from the medial plica and pannus tissue of stage IV OA knees. (a-f) Cells were treated for 48 h with 50 ng/ml of IL-1β (a-c) or 100 ng/ ml of TNF-α (d-f), then MMP levels were measured in the culture supernatant by ELISA. (g-l) Cells were treated for 48 h with 50 ng/ml of IL-1β (g-i) or 100 ng/ ml of TNF-α (j-l), then TIMP levels were measured in the culture supernatant by ELISA. Each bar represents the mean ± standard deviation for 3 separate experiments (* P < 0.05 versus control).

The effect of IL-1β and TNF-α on MMP-2, MMP-3, MMP-9, TIMP-1, TIMP-2, and TIMP-4 release by cultured cells isolated from plica and pannus-like tissue of stage IV OA knees was then examined by ELISA. As shown in Figure 5, stimulation with 50 ng/ml of IL-1β for 48 h resulted in a significant increase in MMP-2 and MMP-3 levels, but not MMP-9 levels, in the medium from both types of cell (panels a-c), whereas stimulation with 100 ng/ml of TNF-α for 48 h resulted in a significant increase in MMP-9 levels in the medium of cells from plica (panel f), a significant decrease in MMP-2 levels for both types of cells (panel d), and no effect on MMP-3 levels (panel e). In terms of TIMP release, both treatments resulted in a significant increase in TIMP-2 levels in the medium from cells from pannus-like tissues, but not those from the plica (panel h), while neither had any effect on TIMP-1 (panel g) or TIMP-4 (panel i) levels.

### Effect of IL-1β or TNF-α on the migration of cells from plica and pannus-like tissue from stage IV patients

**Figure 6:**
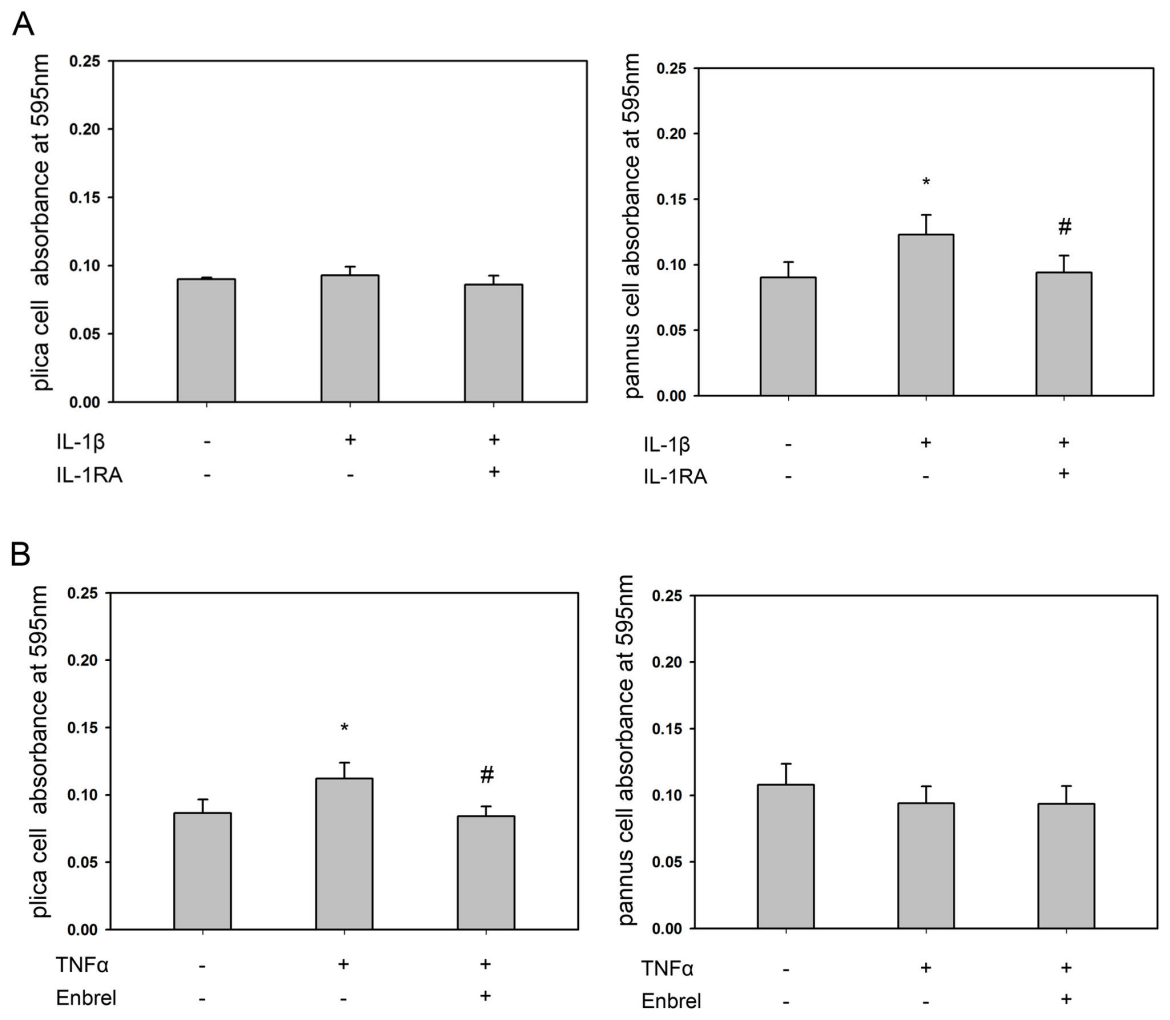
Migration assay of cells from medial plica and pannus-like tissue after treatment with IL-1β or TNF-α with or without a cytokine inhibitor. The medial plica (left panels) and pannus (right panels) cells were treated for 24 h with (A) medium or 50 ng/ml of IL-1β either alone or together with 100 ng/ml of IL-1β antagonist (RA) or (B) medium or 100 ng/ml of TNF-α either alone or together with 100 ng/ml of Enbrel^®^, then the cells were fixed and stained with crystal violet and the OD_595_ measured. Each bar represents the mean ± standard deviation for 6 separate experiments (* p < 0.05 versus control, #p < 0.05 versus cytokine alone).

To examine the effect of IL-1β or TNF-α on the migratory abilities of cells isolated from plica and pannus-like tissue, the cells were seeded into the upper chamber of a porous membrane cell culture insert and allowed to migrate for 24 h across the membrane in the presence or absence of IL-1β or TNF-α or in the presence of IL-1β and IL-1 receptor antagonist (IL-1 RA) or TNF-α and the TNF-α inhibitor Enbrel. As shown in Figure 6A, IL-1β had no effect on the migration of plica-derived cells (left panel), but stimulated the migration of pannus-like tissue-derived cells and this effect was blocked by IL-1RA (right panel), while, as shown in Figure 6B, TNF-α had no effect on pannus-like tissue cell migration (right panel), but stimulated the migration of plica cells and this effect was blocked by Enbrel (left panel). These data show that IL-1β can stimulate pannus-like cell migration and TNF-α can stimulate plica cell migration.

## Discussion

In this study, we demonstrated that, in the medial plica and pannus-like tissue of the knees of patients with stage IV medial compartment OA, (i) levels of IL-1ß and TNF-α mRNAs were significantly higher than in normal cartilage, (ii) MMP-2, MMP-3, MMP-9, TIMP-1, TIMP-2, and TIMP-4 mRNA levels were significantly higher than in normal cartilage and immunohistochemical staining demonstrated expression of these MMPs and TIMPs in the plica and pannus-like tissue, (iii) MMP-2, MMP-3, MMP-9, TIMP-1, TIMP-2, and TIMP-4 protein levels were higher than in stage II OA, (iv) MMP-2 and MMP-3 release by cells isolated from both tissues was increased after IL-1ß treatment, MMP-9 release from both cell types was increased after TNF-α treatment, and TIMP-2 release from pannus-like tissue cells was increased after either treatment, and (v) the migration of cells isolated from pannus-like tissue or plica was increased, respectively, after IL-1β treatment or TNF-α treatment. 

The mediopatellar plica is a collagen-rich fibrous tissue that extends from the synovial capsule of the knee and abrasion or impingement between the medial plica and the opposing medial femoral condyle has been described in patients with medial compartment OA of the knee [15,20,21,26,27]. A later study [28] found that the repeated injuries elicited by this abrasion can trigger IL-1ß production, thus increasing MMP-3 expression, and demonstrated expression of IL1-ß mRNA and MMP-3 mRNA and protein in the medial plica of early stage OA knees, suggesting that this structure and its interplay with the opposing medial femoral condyle might play a role in the pathogenesis of the medial compartment OA knee. 

Pannus-like tissue, an invasive granulation tissue, has been found in the knees of patients with advanced OA [36,46]. The observed IL-1ß and MMP-3 protein expression in this tissue [36,46] suggests that these proteins might be involved in the pathogenesis of OA. Recently, pannus-like tissue was also discovered on the cartilage of the medial femoral condyle opposite the inflamed medial plica in early stage medial compartment OA of the knee [26,27] and another study [28] showed that MMP-3 mRNA and protein are highly expressed in pannus-like tissue and that the cells isolated from this tissue show increased MMP-3 mRNA levels after IL-1ß treatment. In the present study, we detected high levels of IL-1β and TNF-α protein in medial plica and pannus-like tissue of the OA knee, providing more evidence that these tissues may play important roles in the pathogenesis of medial compartment OA of the knee.

High protein levels of MMP-2, MMP-3, and MMP-9 have been found in OA cartilage [47] and subchondral bone [48]. In the present study, both mRNA and protein levels of MMP-2, MMP-3, MMP-9, TIMP-1, TIMP-2, and TIMP-4 in both medial plica and pannus-like tissue were higher in stage IV OA than in stage II OA. Moreover, levels of mRNAs for these six proteins were also higher in medial plica and pannus-like tissue of stage IV OA patients than in stage II patients. TIMPs regulate ECM remodeling by inhibiting MMP function [39] and overexpression of MMPs results in an imbalance between MMPs and TIMPs activities that can lead to pathological disorder [49] or tissue degradation [50]. In OA, TIMP expression does not increase to the same extent as MMP expression and this imbalance may contribute to cartilage breakdown [39]. 

We found that the ratio of TIMP-1, TIMP-2, or TIMP-4 to MMP-2 or MMP-3 and the TIMP-2/MMP-9 ratio decreased significantly going from stage II to stage IV OA, demonstrating a greater increase in protease activities than in TIMPs levels as medial compartment OA progressed. The imbalance between these three TIMPs and MMPs in the medial plica of OA knees may contribute to cartilage destruction.

The mechanical abrasion of the medial femoral condyle by the medial plica during knee motion causes various degrees of inflammation in the medial plica [26]. The present study revealed that high levels of mRNAs for the primary inflammatory cytokines IL-1β and TNF-α were detected in plica and pannus-like tissues of knee OA patients. We found that these two cytokines upregulated MMPs and TIMPs to different degrees, resulting in a TIMP/MMP imbalance, which may lead to the destruction of articular cartilage. Our in vitro migration assay further demonstrated that both IL-1β-treated pannus-like cells and TNFα-treated plica cells increased their migration ability. MMP upregulation [44] and increased synovial cell migration [45] have also been reported in rheumatoid arthritis. Our results suggest that, as in rheumatoid arthritis, the IL-1β- and TNF-α-rich pannus-like tissue may be an invasive tissue involved in the progression of knee osteoarthritis. 

In conclusion, the results of our studies suggest that medial plica and pannus-like tissue may play roles in the process of cartilage degradation in OA knee by producing ECM-degrading enzymes during inflammation and that the imbalance between TIMP and MMP levels in these tissues increases protease activity in the medial compartment and this may also contribute to cartilage breakdown and progression of osteoarthritis.
